# Asthma and COPD exacerbation in relation to outdoor air pollution in the metropolitan area of Berlin, Germany

**DOI:** 10.1186/s12931-022-01983-1

**Published:** 2022-03-20

**Authors:** Christina Hoffmann, Mariam Maglakelidze, Erika von Schneidemesser, Christian Witt, Peter Hoffmann, Tim Butler

**Affiliations:** 1 Department of Outpatient Pneumology, Charité – Universitätsmedizin Berlin, corporate member of Freie Universität Berlin and Humboldt-Universität zu Berlin, Augustenburger Platz 1, 13353 Berlin, Germany; 2 Institute for Advanced Sustainability Studies e.V. (IASS), Potsdam, Germany; 3 Petre Shotadze Tbilisi Medical Academy, Tbilisi, Georgia

**Keywords:** Nitrogen dioxide, Ozone, Particulate matter, Hospital admission, Morbidity, Limit values

## Abstract

**Background:**

Ambient air pollution poses a major risk for the development and aggravation of respiratory diseases. Evidence suggests that even in low-level air pollution environments there is a risk for an increase in adverse respiratory symptoms. We examined whether variations in daily air pollution levels of nitrogen dioxide, ozone, or particulate matter in Berlin, Germany were associated with hospital admissions of chronic obstructive pulmonary disease (COPD) and asthma patients in a time series analysis.

**Methods:**

We calculated single and multi-pollutant models, investigated possible lags in effect, and analysed the influence of meteorological variables on the results. Data from January 2005 through December 2015 were used to quantify the concentration–response.

**Results:**

The risk ratio for asthma patients to be hospitalised on the same day of NO_2_ exposure was 1.101 per 10 µg/m^3^ NO_2_ increase (95% CI: 1.013 to 1.195), for COPD patients 1.123 (95% CI: 1.081 to 1.168). Neither the exposure to ozone (95% CI: 0.904 to 1.020), PM_10_ (95% CI: 0.990 to 1.127), nor PM_2.5_ (95% CI: 0.981 to 1.148) was associated with an increased risk ratio for asthma patients to be hospitalised**.** Risk ratios for the hospital admission of COPD patients were also not increased due to ozone (95% CI: 0.981 to 1.033), PM_10_ (95% CI: 0.988 to 1.032), or PM_2.5_ (95% CI: 0.966 to 1.019) exposure. The presented risk ratios and confidence intervals relate to the day of exposure. We found no increased hospitalisation risks with a delayed occurrence on subsequent days.

**Conclusions:**

A quantifiable, statistically significant increase in risk for asthma and COPD exacerbations owing to NO_2_ exposure at levels well below European regulatory limit values was observed.

**Supplementary Information:**

The online version contains supplementary material available at 10.1186/s12931-022-01983-1.

## Background

Ambient air pollution poses a major risk for the development and aggravation of respiratory diseases. Positive associations between short-term exposures to particulate matter (PM with an aerodynamic diameter of 2.5 µm or less, PM_2.5_), sulphur dioxide (SO_2_), nitrogen dioxide (NO_2_) and health endpoints related to chronic obstructive pulmonary disease (COPD) morbidity and mortality have been observed [1]. Adverse effects of short-term ozone (O_3_) exposure on respiratory health endpoints have also been reported [2, 3]. Air pollution was shown to be associated with the development of asthma and morbidity among children [4]. Evidence suggests that even in low-level air pollution environments, there is a risk for an increase in adverse respiratory symptoms in adults and children; exposure to traffic and related pollutants is associated with decreased lung function and the onset of asthma [5–9]. Relative risk estimates from air pollution studies vary, since they use different increment units and target groups for analysis. World Health Organization (WHO) Air Quality Guidelines suggested that relative risks for various respiratory health endpoints associated with nitrogen dioxide led to a 1.4–3.3% increase in hospitalisation or hospital visits per 10 µg/m^3^ (24 h) increase [10].

In Germany, one of the main sources of air pollutant emissions is the transportation sector [11]. Despite the policies implemented in recent years to ameliorate the air pollution situation in Berlin, NO_2_ and PM_10_ concentrations still exceed the limit values in certain areas of the city, posing health risks [11]. While European ambient air quality standards apply to NO_2_, emission limit values for vehicles are specified as emissions of NO_x_ (the sum of NO and NO_2_). Estimates of the fraction of NO_x_ emitted as NO_2_ vary between 15 and 25% [12]. Much of the NO_2_ measured at urban background locations is due to secondary production through reaction of NO with O_3_, a reversible process which simultaneously generates NO_2_ and removes O_3_ [13]. The reversible nature of this process often leads to higher observed ozone concentrations under conditions of low NO_x_ in urban areas [14]. In short, the concentrations of NO_2_ and O_3_ are strongly linked.

Few studies have been conducted in Germany, examining the relation of air pollution and health. A study on behalf of the German Environment Agency demonstrated that in 2014, background concentrations of NO_2_ appeared to be detrimental for several non-communicable diseases and mortality in Germany [15]. However, a study in Hamburg found that respiratory emergency department visits were significantly associated with temporal variables, but environmental variables showed no direct associations [16]. Interactive effects between equivalent temperature and air pollution on mortality for Berlin and Lisbon have also been demonstrated [17]. Air pollution as a risk factor of respiratory morbidity has not been researched for Berlin. In our study, we aimed to identify and quantify associations between short-term exposure to air pollutant concentrations and respiratory health endpoints in Berlin. The objective of the study was to examine whether daily air pollution variations of the pollutants NO_2_, O_3_, PM_10_, and PM_2.5_ were associated with hospital admissions of COPD and asthma patients, and if so, to quantify the concentration–response. We analysed urban background pollutant concentrations that are characteristic of the urban area as a whole, independent of local hot-spots [11].

## Methods

### Study design

The study was designed as a retrospective time series analysis. The ethics committee of the Charité-Universitätsmedizin Berlin approved the project (EA2/147/17). We performed the study according to the principles of the Declaration of Helsinki.

The study population consisted of patients hospitalised due to an acute exacerbation of COPD or asthma. Trained physicians diagnosed the diseases according to the current guidelines: COPD diagnosis was based on post-bronchodilator spirometry results, as recommended by the Global Initiative for Chronic Obstructive Lung Disease (GOLD) [18]. For the diagnosis of asthma, the guidelines of the Global Initiative for Asthma (GINA) were applied [19]. COPD patients with an age below 40 years and asthma patients younger than 18 years were excluded from the study.

### Study setting

**Fig. 1:**
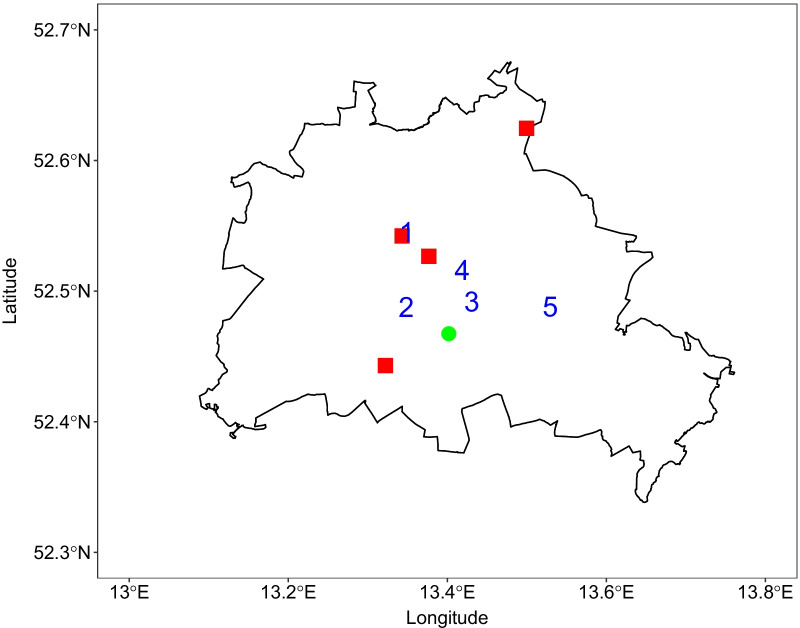
City of Berlin boundaries (black line) with Charité campuses (red squares), meteorological station (green dot), and urban background air quality monitoring stations (blue numbers). NO_2_ data was available at all five stations, PM at stations 1, 3, and 4, O_3_ at stations 1 and 3

The patient data originated from the Charité-Universitätsmedizin Berlin, a university hospital with four campuses across Berlin, shown in Fig. 1. Within the Charité, 3,000 hospital beds are available for the treatment of about 150,000 inpatients and 690,000 outpatients per year.

### Data collection and endpoints

#### Hospital admission data

The primary endpoint of the study was the daily number of hospital admissions for COPD or asthma exacerbation. We retrieved the admission data from the digital hospital information system. Admissions between January 1st, 2005 to December 31st, 2015 were included from all Charité hospitals in Berlin. To qualify for inclusion, cases had to be labelled with the International Statistical Classification of Diseases and Related Health Problems, 10th Revision, German Modification (ICD-10-GM) codes J44 (COPD) or J45 to J46 (asthma) in the main or secondary diagnoses. If codes J44 to J46 were part of the secondary diagnoses, the main diagnosis had to be J09 to J11 (influenza), J12 to J18 (pneumonia), J20 or J22 (other acute infection of the lower respiratory tract), J40 to J43, or J47 (chronic disease of the lower respiratory tract). Smoking status was identified by the presence or absence of ICD code F17 (mental and behavioural disorders due to use of tobacco).

#### Air pollution data

Air pollution data including NO_2_, O_3_, PM_10_, and PM_2.5_ were retrieved from the Berlin city regulatory air quality monitoring network [20]. The network of measuring stations is run by Berlin’s Senate Department for the Environment, Transport, and Climate Protection. Within the network, NO_2_ was measured by chemiluminescence, O_3_ by ultra-violet absorption, and PM by sequential filter sampling, conforming to EU standards. The daily concentration values originated from five urban background stations for NO_2_, three stations for PM_10_ and PM_2.5_, and two stations for O_3_, marked in Fig. 1. The values from all available urban background stations were averaged to calculate one daily concentration value for each pollutant. The total oxidants (O_x_) concentration was calculated as the sum of nitrogen dioxide and ozone concentrations.

#### Meteorological data

The daily mean temperature, average humidity and wind speed were obtained from a meteorological station at the former Berlin-Tempelhof airport, available online through the website of the German Meteorological Office (Deutscher Wetterdienst, www.dwd.de). The meteorological station is located in the urban area of Berlin at 48 m above sea level on 52.47°N and 13.40°E, see Fig. 1.

### Statistical analysis

The statistical analysis was performed with IBM SPSS Statistics version 25 (IBM Corporation, Armonk, NY, USA) and R: A language and environment for statistical computing [21]. A common method in environmental epidemiology to analyse the effects of air pollutants or meteorological parameters on a population’s health is a time series regression [22]. For the time series analysis, we used the distributed lag non-linear model (dlnm) package created by Gasparrini [23]. The method serves to calculate risk ratios: the probability of an outcome (e.g. hospitalisation) after a certain exposure (e.g. air pollutant concentration). If the risk ratio (RR) is greater than 1, the probability of the outcome is increased by the exposure. Furthermore, the dlnm allows the consideration of lag days, the delayed occurrence of health effects after exposure. We calculated the risk ratios for the day of exposure and the following 7 (lag) days. All lag terms were modelled together in an unconstrained dlnm. For the generalised linear model, a quasi-Poisson distribution was assumed. When creating the cross-basis, which specifies the exposure-lag-response dependency, we chose a linear function to model the relationship for air pollutants and strata for the ambient temperature. The variable for lag days was defined as integer values. A natural cubic spline of time with 7 degrees of freedom per year (76 in total) was created to control for seasonal and long-term trends. First, single pollutant models were calculated, including the mean temperature. If the model showed statistical significance, the remaining meteorological variables wind speed and humidity were added one by one. The single pollutant models with statistical significance were then combined into multi-pollutant models. P-values less than 0.05 were considered statistically significant.

### Sample size calculations

The precision of time series analyses increases with the length of the series and the number of events per day. For studies on the effects of air pollutants, several thousands of observation days are recommended [22]. We analysed a time series of 4,017 days.

## Results

The characteristics of the asthma and COPD patients included into the study are reported in Table 1. The majority of the asthma patients were female (61.1%), while the COPD cohort consisted of more male patients (58.3%). Asthma and COPD patients had a mean age of 52 and 68 years, respectively. Only 4.9% of the asthma and 8.3% of the COPD patients were current smokers. A minor percentage of the admitted patients were infected with influenza.

**Table 1:** Description of patient cohorts

Parameter	Asthma	COPD
Admissions, n	876	8645
Male, n (%)	341 (38.9%)	5038 (58.3%)
Female, n (%)	535 (61.1%)	3607 (41.7%)
Age, years, mean ± SD	52.2 ± 19.5	68.1 ± 10.2
Current smoker, n (%)	43 (4.9%)	721 (8.3%)
Influenza, n (%)	8 (0.9%)	20 (0.2%)
Length of stay, days, mean ± SD	7.5 ± 8.8	10.9 ± 14.2
Died in hospital, n (%)	15 (1.7%)	392 (4.5%)

Table 2 summarizes the range of air pollutant and meteorological variable values. The median pollutant concentrations were all below the limit values set by the WHO Air Quality Guidelines, as well as by the EU Air Quality Directive [10, 24].

**Table 2:** Median and range of daily air pollutant concentrations and meteorological variables

Parameter	Median (range)
NO_2_ [µg/m^3^]	25.0 (6.0–87.0)
O_3_ [µg/m^3^]	42.0 (1.0–135.0)
O_x_ [µg/m^3^]	68.0 (20.5–157.0)
PM_10_ [µg/m^3^]	21.7 (4.7–188.3)
PM_2.5_ [µg/m^3^]	15.5 (3.8–168.8)
Daily mean temperature [°C]	10.8 (− 15.6–30.5)
Humidity [%]	76.38 (0.00–99.96)
Wind speed [m/s]	35.0 (0.0–106.0)

**Fig. 2:**
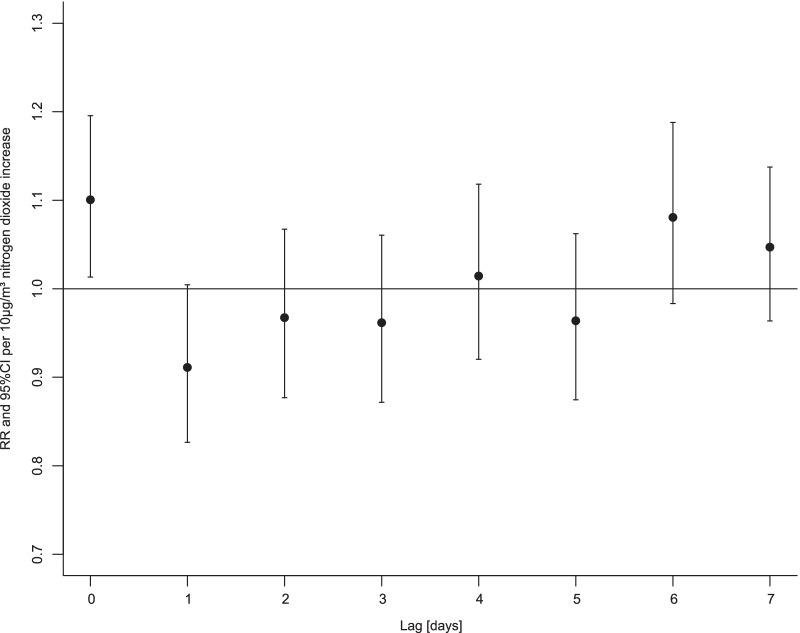
Hospitalisation risk ratios of asthma patients after NO_2_ exposure Displayed are risk ratios (RR, dots) and 95% confidence intervals (CI, whiskers) per 10 µg/m^3^ increase in nitrogen dioxide concentration

Plots of NO_2_, O_3_, PM_10_, and PM_2.5_ concentrations over time can be found in Additional file 1: Fig. S1–S4. In the single pollutant model, the risk ratio for asthma patients to be hospitalised on the same day of NO_2_ exposure was 1.101 per 10 µg/m^3^ NO_2_ increase (95% confidence interval, CI: 1.013 to 1.195). There were no lagged effects seven days after exposure, as shown in Fig. 2. Neither the exposure to ozone (95% CI: 0.904 to 1.020), total oxidants (95% CI: 0.962 to 1.117), PM_10_ (95% CI: 0.990 to 1.127), nor PM_2.5_ (95% CI: 0.981 to 1.148) was associated with an increased risk ratio for asthma patients to be hospitalised. The listed confidence intervals refer to the day of exposure. No multi-pollutant model showed statistical significance.

**Fig. 3:**
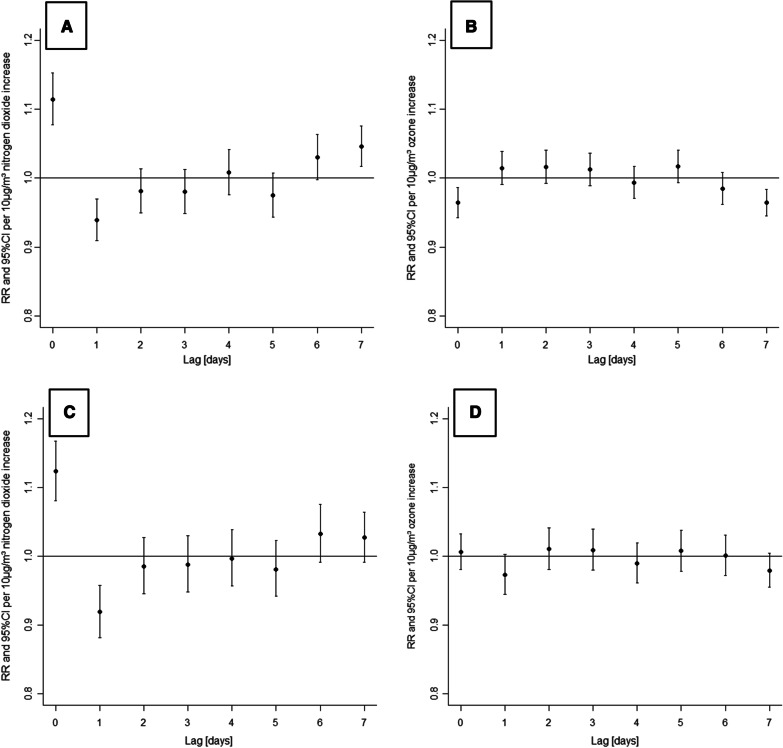
Hospitalisation risk ratios of COPD patients after exposure to NO_2_ and ozone (single and multi-pollutant modelling results). Panel **A** (NO_2_) and **B** (O_3_) show the single pollutant modelling results, including mean temperature and wind speed. The results of the multi-pollutant model combining exposure to NO_2_, ozone, mean temperature, and wind speed are given in panels **C** (NO_2_) and **D** (O_3_). Displayed are risk ratios (RR, dots) and 95% confidence intervals (CI, whiskers) per 10 µg/m^3^ increase in air pollutant concentration

The single pollutant models for NO_2_ and ozone showed significantly changed risk ratios for COPD patients. In both models, wind speed had a significant risk increasing effect (NO_2_: p < 0.001, O_3_: p = 0.035). NO_2_ exposure was associated with an increased risk ratio for a disease exacerbation on the same day (Fig. 3A, 1.114 per 10 µg/m^3^ NO_2_ increase, 95% CI: 1.077 to 1.152), while exposure to ozone was linked to decreased risk ratios (see Fig. 3B). No change in risk ratios was observed due to PM_10_ exposure (95% CI: 0.988 to 1.032) or exposure to PM_2.5_ (95% CI: 0.966 to 1.019).

When the models for NO_2_ and ozone were combined to one multi-pollutant model, there was no longer a decreased risk ratio for COPD patients to be hospitalised due to ozone exposure (95% CI: 0.981 to 1.033 on the day of exposure, Fig. 3D). The risk ratio for COPD patients to be hospitalised on the same day of NO_2_ exposure was 1.123 per 10 µg/m^3^ NO_2_ increase (95% CI: 1.081 to 1.168).

To confirm the results of the multi-pollutant model, we analysed a time series with the total oxidants (O_x_) concentrations, consisting of the added NO_2_ and O_3_ values. Exposure to O_x_ also showed an increased risk ratio of 1.030 per 10 µg/m^3^ O_x_ increase for a hospitalisation on the day of exposure (95% CI: 1.005 to 1.056), as shown in Additional file 1: Fig. S5.

## Discussion

Our results indicate that in Berlin, at relatively low levels of air pollutant concentrations there is an increased risk of COPD and asthma exacerbations leading to hospitalisation. Effects on hospitalisations of COPD patients were larger with higher wind speed. Compared to other pollutants (PM_10_, PM_2.5_, ozone), only NO_2_ was associated with an increased risk. The NO_2_ concentration increased the risk for asthma patients to be hospitalised on the day of exposure by 10% per 10 µg/m^3^ NO_2_ increase. Our multi-pollutant model showed that only NO_2_ contributed to an increased morbidity associated with COPD hospitalisation on the day of increase of pollutant concentration (by 12% per 10 μg/m^3^ NO_2_). The increased O_3_ concentrations did not offset the benefits of lower NO_2_ concentrations. We note that the analysis presented here is based on urban background measurements of NO_2_, which are lower than the European limit values for ambient concentration, and likely a lower bound for exposure.

Our analysis resulted in higher effect estimates per 10 µg/m^3^ increase of NO_2_ on asthma and COPD patients than described in other studies. Respiratory disease hospitalisations in relation to NO_2_ have been reported by studies in Iran [25] and China [26], however, with lower risk estimates compared to our results. Similar to this study, Gao et al. observed short-term effects of ambient air pollution on COPD hospitalisation, with NO_2_ showing the most pronounced effect, compared to other gaseous pollutants and particles [27]. The results of our study compared to some European studies are similar: relative to PM_10_, NO_2_ had a stronger effect on multiple respiratory events in six Italian cities. Effects were instantaneous (lag 0–1) and mostly expressed with regard to COPD [28]. Gaseous air pollutants were shown to be important factors for acute hospitalisations for respiratory health endpoints in a time series analysis from Rome [29]. Furthermore, a systematic review by Mills et al. reported associations between NO_2_ and adverse health outcomes that were independent of PM mass [30].

Conflicting results with regard to the delayed effects on respiratory hospitalisations have been reported in other studies: Szyszkowicz et al. found that NO_2_ was positively associated with COPD emergency visits for males 3–6 days after exposure and for females after 8 lag days [9]. Effects of air pollution on respiratory hospital admissions in Turkey showed that the relative risk was highest at lag day 4 [31]. These inconsistencies could be due to variations in study designs, sample sizes, disease prevalence, cohort characteristics, access to healthcare, air pollution concentrations, geographical differences, and availability of air pollution data. For respiratory patients in Berlin, an immediate hospital admission could be explained by the availability of hospitals in close proximity, with 24-h emergency departments.

The single and multi-pollutant models for the effects of NO_2_ exposure on COPD patients in our study both showed a decreased risk for hospital admission at lag day 1 (Fig. 3A and C). One explanation for this harvesting effect [22] is that on a day with high ambient concentrations of NO_2_, a disease exacerbation is triggered in many vulnerable COPD patients. On the following day, the number of vulnerable COPD patients not already in hospitals is reduced, which leads to fewer exacerbations and a decreased risk ratio for hospitalisation.

We also observed that wind speed played a role in increasing the risk of COPD exacerbations in the presence of air pollution. Environmental and chemical exposures are known to trigger COPD exacerbations [32] and many people with COPD often report difficulties with breathing during cold air and strong winds [33]. Few publications exist on the influence of meteorological factors on COPD and asthma patients in Germany. However, in North Bavaria, a 1% increase in daily ambulatory visits of COPD patients was associated with an increased wind speed. The authors stated that the exact mechanism how strong winds increase COPD morbidity is unclear, but could have complex reasons, as heterogeneous as the pathogenesis of COPD itself [34].

We analysed the effects of air pollution on two cohorts: asthma and COPD patients. Asthma is predominantly an airway disease, while COPD is a progressive disease including emphysema and chronic bronchitis, affecting different age groups (Table 1). Our study design was chosen to investigate the effects of air pollution on the respiratory health of adults of all ages.

Two possible confounding risk factors for exacerbation, smoking and influenza infection, were considered. The prevalence of smoking was low in both cohorts and few patients were admitted with influenza (Table 1). Both single and multi-pollutant models were applied to test the robustness of the NO_2_ results. The analysis of the total oxidants O_x_ (NO_2_ + O_3_) served as additional confirmation and demonstrated that high ozone concentrations do not outweigh the effects of NO_2_.

Our study had several limitations. We did not measure indoor air pollution or personal exposure to pollutants. Nevertheless, the location of the monitoring stations in the city of Berlin, as per EU guidelines, does ensure that the concentrations of air pollutants used in this study are broadly representative of the urban area. We used urban background concentrations in our model which are consistently lower than, for example, concentrations measured next to locations with high traffic. It should be taken into account that we only investigated exacerbations that led to a hospitalisation. COPD and asthma exacerbations not requiring admissions were not included, probably leading to an underestimation of the effects of air pollutants on respiratory outcomes. The applied time series model does not consider demographic, socio-economic, or behavioural variables of the individual participants. This could be an area of further research.

While the observed correlation of NO_2_ and hospital admissions is not definite proof for a causal relationship between NO_2_ exposure and physiological damage, NO_2_ should be seen as an indicator substance for a more complex mixture of pollutants, which is released e.g. by combustion processes in vehicles, airplanes, ships, and factories. Detrimental effects could be caused by other substances of this pollutant mixture that correlated with NO_2_. Chronic and sub-chronic exposure to low levels of NO_2_ is reported to be unfavourable with regard to lung metabolism, function, structure, and even patients’ susceptibility to pulmonary infections [10].

Our results have important policy implications as NO_2_ is a pollutant produced locally with a short lifetime in the atmosphere. In Berlin, NO_2_ emissions from traffic are estimated to be responsible for about 70–80% of the pollution in the inner city residential areas [11]. Acute exacerbations of COPD and asthma, and consequent hospitalisations generate significant costs for the healthcare system and are correlated with a decline in lung function and disease progression [35]. Addressing the risk factors for these adverse events plays an important role, given that some projections show that NO_2_ will be the pollutant most profoundly associated with respiratory hospital admissions in the coming years [36].

## Conclusions

We demonstrated that NO_2_ is the pollutant with the largest risk ratios for hospital admissions of asthma and COPD patients in Berlin. Our models show that NO_2_ contributes to increased morbidity more significantly than PM, and that increased O_3_ typically associated with episodes of lower NO_2_ does not offset the benefits of lower NO_2_ concentrations. Furthermore, the analysis presented here is based on urban background measurements of NO_2_, which are consistently lower than the European limit values for ambient concentrations.

## Supplementary Information

**Additional file 1: Figure S1.** Urban background concentrations of nitrogen dioxide over the study period. **Figure S2.** Urban background concentrations of ozone over the study period. **Figure S3.** Urban background concentrations of PM_10_ over the study period. **Figure S4.** Urban background concentrations of PM_2.5_ over the study period. **Figure S5.** Hospitalisation risk ratios of COPD patients after exposure to total oxidants (O_x_).

## Data Availability

The datasets generated and/or analysed during this study are available from the corresponding author on reasonable request.

## Abbreviations

COPD: Chronic obstructive pulmonary disease
NO_2_: Nitrogen dioxide
CI: Confidence interval
PM_10_: Particulate matter with an aerodynamic diameter of 10 µm or less
PM_2.5_: Particulate matter with an aerodynamic diameter of 2.5 µm or less
SO_2_: Sulphur dioxide
O_3_: Ozone
WHO: World Health Organization
NO_x_: The sum of nitrogen oxide and nitrogen dioxide concentrations
NO: Nitrogen oxide
GOLD: Global Initiative for Chronic Obstructive Lung Disease
GINA: Global Initiative for Asthma
ICD-10-GM: International Statistical Classification of Diseases and Related Health Problems, 10th Revision, German Modification
EU: European Union
O_x_: Total oxidants, the sum of nitrogen dioxide and ozone concentrations
dlnm: Distributed lag non-linear model
RR: Risk ratio
SD: Standard deviation
