# Supplementary material for: Asthma and COPD exacerbation in relation to outdoor air pollution in the metropolitan area of Berlin, Germany
Source: Respir Res. 2022 Mar 20;23:64. doi: 10.1186/s12931-022-01983-1 (PMC8935815; doi:10.1186/s12931-022-01983-1)
Supplement: Supplementary file 1 — Additional file 1: Figure S1. Urban background concentrations of nitrogen dioxide over the study period. Figure S2. Urban background concentrations of ozone over the study period. Figure S3. Urban background concentrations of PM10 over the study period. Figure S4. Urban background concentrations of PM2.5 over the study period. Figure S5. Hospitalisation risk ratios of COPD patients after exposure to total oxidants (Ox). [file 12931_2022_1983_MOESM1_ESM.pdf]

Supplementary Information for

Asthma and COPD Exacerbation in Relation to Outdoor Air Pollution in the Metropolitan Area of Berlin, Germany

Christina Hoffmann[1a], Mariam Maglakelidze[2,3a], Erika von Schneidemesser[2], Christian Witt[1], Peter Hoffmann[1b], Tim Butler[2b]

[a] both first authors contributed equally to the manuscript

[b] both last authors contributed equally to the manuscript

[1] Charité – Universitätsmedizin Berlin, corporate member of Freie Universität Berlin and Humboldt-Universität zu Berlin, Department of Outpatient Pneumology, Berlin, Germany

[2] Institute for Advanced Sustainability Studies e.V. (IASS), Potsdam, Germany

[3] Petre Shotadze Tbilisi Medical Academy, Tbilisi, Georgia

Correspondence:
Dr. Christina Hoffmann, Charité – Universitätsmedizin Berlin, Augustenburger Platz 1, 13353 Berlin, Germany.
E-mail: christina.hoffmann2@charite.de, Phone: +49 (0)30 450669034

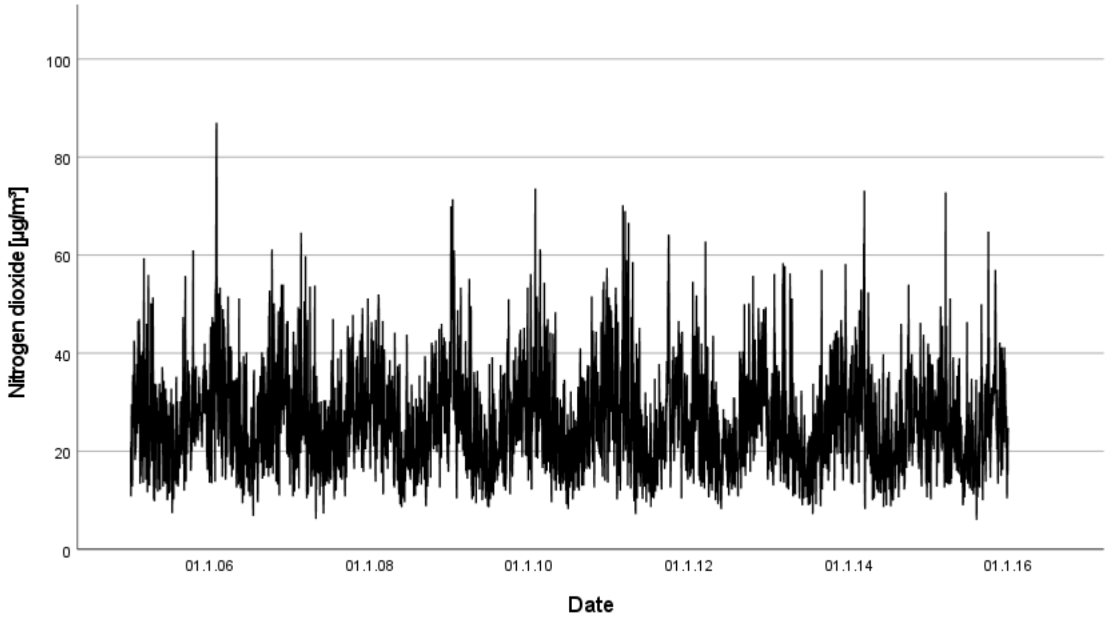

Figure S1. Urban background concentrations of nitrogen dioxide over the study period.

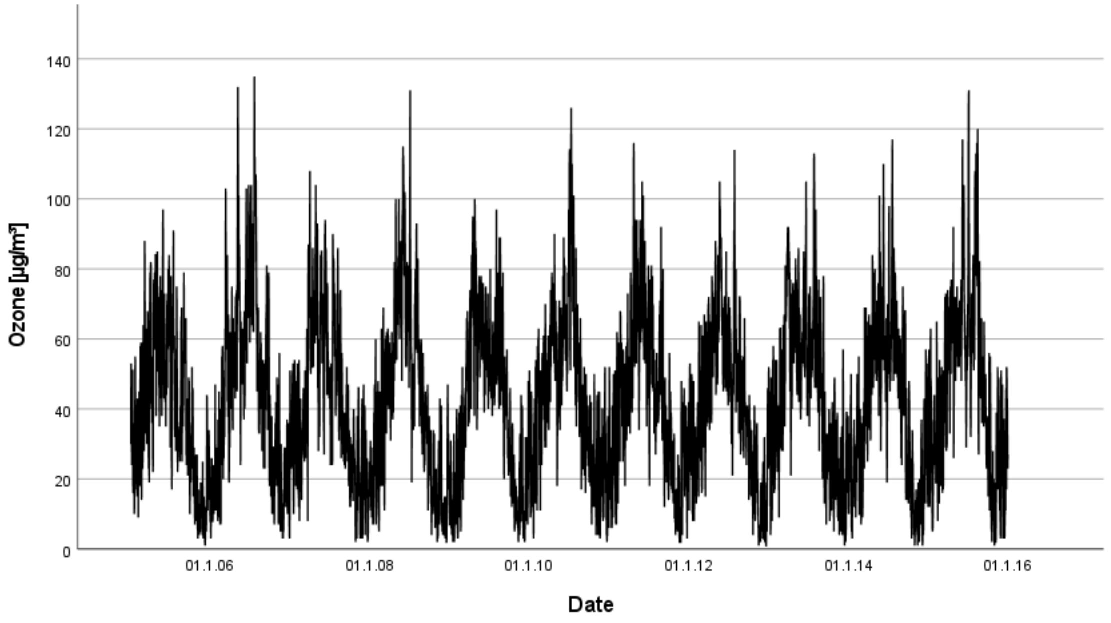

Figure S2. Urban background concentrations of ozone over the study period.

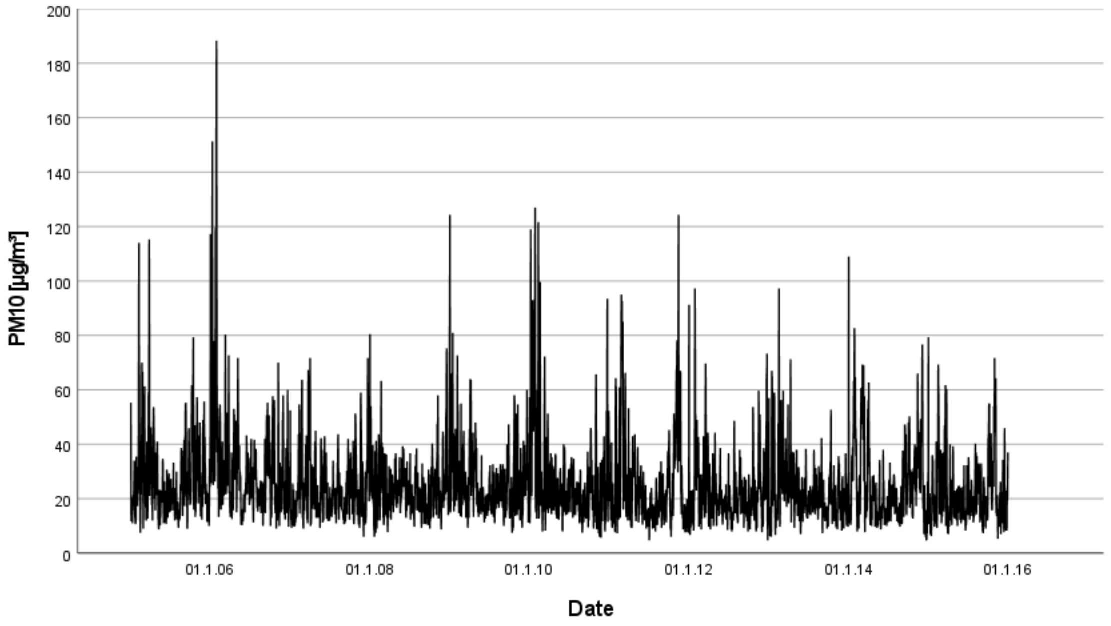

Figure S3. Urban background concentrations of PM_{10} over the study period.

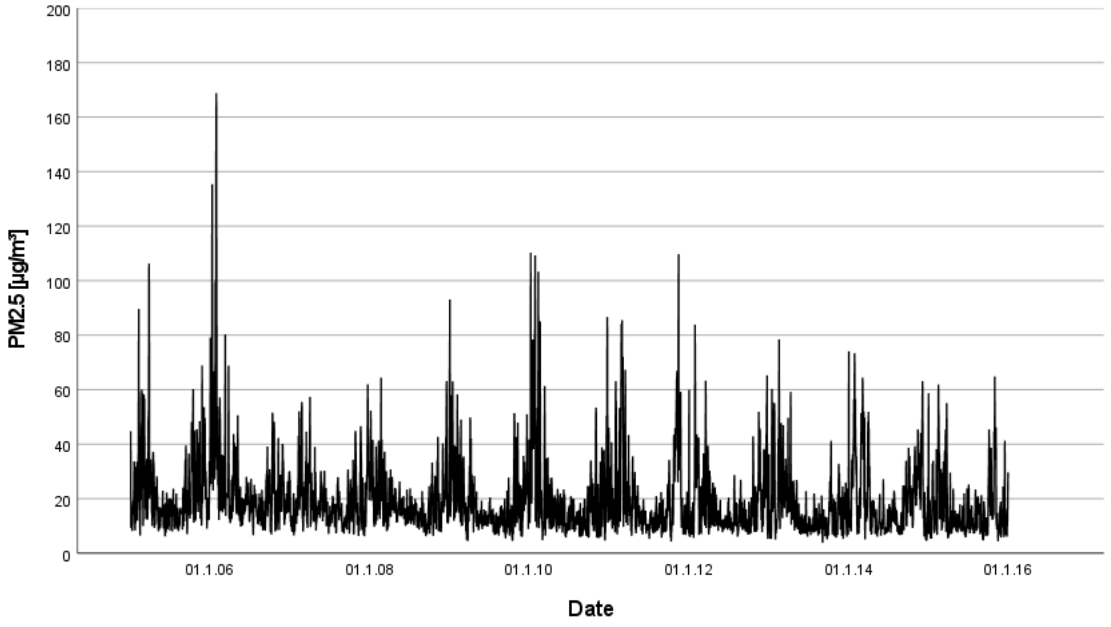

Figure S4. Urban background concentrations of $PM_{2.5}$ over the study period.

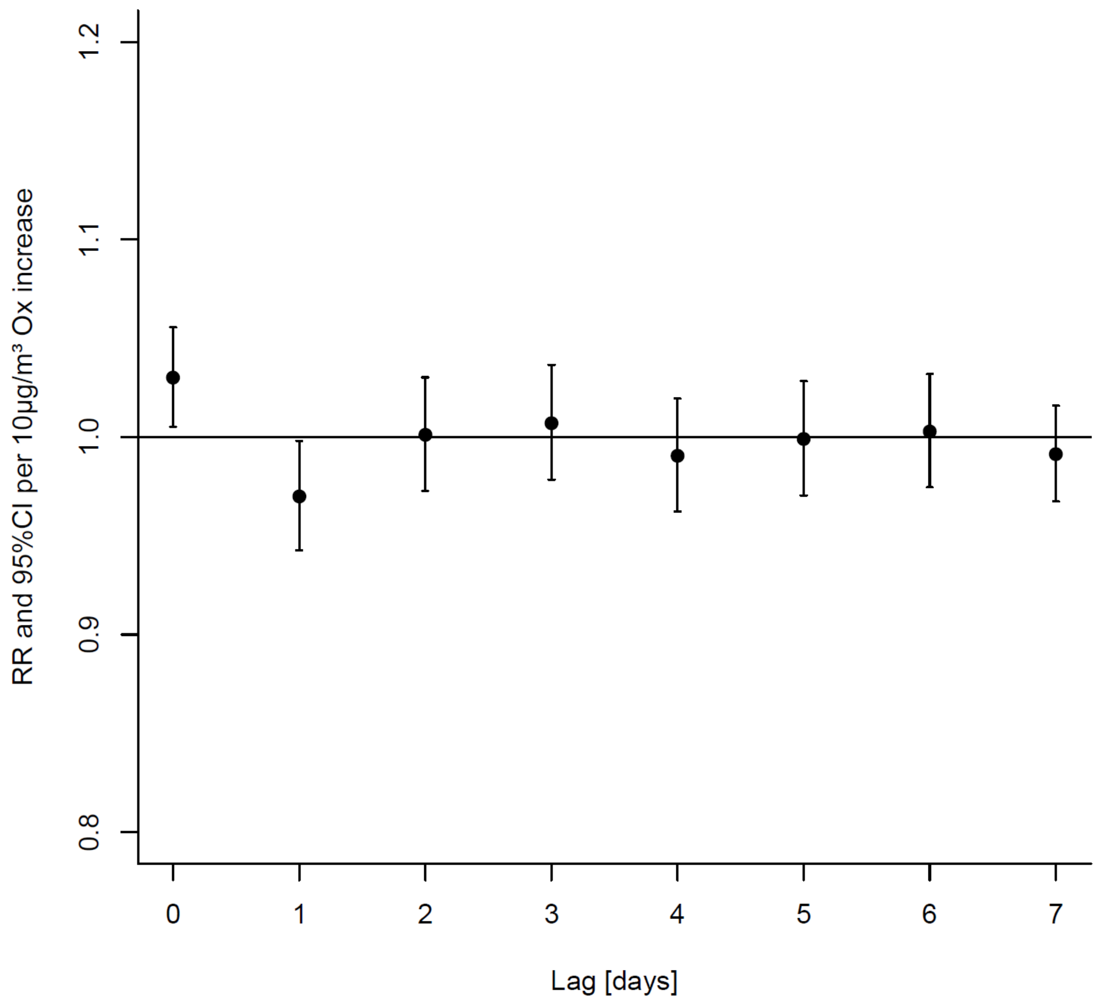

Figure S5. Hospitalisation risk ratios of COPD patients after exposure to total oxidants (O_x). The plot shows the results from a model including the mean temperature. Displayed are risk ratios (RR, dots) and 95% confidence intervals (CI, whiskers) per 10 µg/m³ increase total oxidants concentration.
